# Patterns of Domain-Specific Learning Among Medical Undergraduate Students in Relation to Confidence in Their Physiology Knowledge: Insights From a Pre–post Study

**DOI:** 10.3389/fpsyg.2021.562211

**Published:** 2022-02-10

**Authors:** Jochen Roeper, Jasmin Reichert-Schlax, Olga Zlatkin-Troitschanskaia, Verena Klose, Maruschka Weber, Marie-Theres Nagel

**Affiliations:** ^1^Department of Neurophysiology, University Hospital Frankfurt, Frankfurt, Germany; ^2^Department of Business and Economics Education, Johannes Gutenberg University Mainz, Mainz, Germany

**Keywords:** domain learning, physiology knowledge, knowledge development, confidence testing, learning profiles, medical education, longitudinal study

## Abstract

**Research Focus:**

The promotion of domain-specific knowledge is a central goal of higher education and, in the field of medicine, it is particularly essential to promote global health. Domain-specific knowledge on its own is not exhaustive; confidence regarding the factual truth of this knowledge content is also required. An increase in both knowledge and confidence is considered a necessary prerequisite for making professional decisions in the clinical context. Especially the knowledge of human physiology is fundamental and simultaneously critical to medical decision-making. However, numerous studies have shown difficulties in understanding and misconceptions in this area of knowledge. Therefore, we investigate (i) how preclinical medical students acquire knowledge in physiology over the course of their studies and simultaneously gain confidence in the correctness of this knowledge as well as (ii) the interrelations between these variables, and (iii) how they affect the *development* of domain-specific knowledge.

**Method:**

In a pre–post study, 169 medical students’ development of physiology knowledge and their confidence related to this knowledge were assessed *via* paper-pencil questionnaires before and after attending physiology seminars for one semester. Data from a longitudinal sample of *n* = 97 students were analyzed using mean comparisons, regression analyses, and latent class analyses (LCAs). In addition, four types of item responses were formed based on confidence and correctness in the knowledge test.

**Results:**

We found a significant and large increase in the students’ physiology knowledge, with task-related confidence being the strongest predictor (apart from learning motivation). Moreover, a significantly higher level of confidence at t2 was confirmed, with the level of prior confidence being a strong predictor (apart from knowledge at t2). Furthermore, based on the students’ development of knowledge and confidence levels between measurement points, three empirically distinct groups were distinguished: knowledge gainers, confidence gainers, and overall gainers. The students whose confidence in incorrect knowledge increased constituted one particularly striking group. Therefore, the training of both knowledge and the ability to critically reflect on one’s knowledge and skills as well as an assessment of their development in education is required, especially in professions such as medicine, where knowledge-based decisions made with confidence are of vital importance.

## Relevance and Research Questions

Working in the field of medicine is all about making well-informed decisions and the focus in recent years has shifted—at least in theory—from eminence- to evidence-based frameworks (Cate et al., 2018; Custers and Cate, 2018). In the last 25 years, the principles of evidence-based medicine have been integrated into many core curricula of medical education and also drive current initiatives to improve these curricula (e.g., German National Competence-based Learning Objectives Catalog for Undergraduate Medical Education; Fritze et al., 2017a,b). While there is an agreement about the need for further solid scientific groundwork in the domain of medicine in general, the implications for the individual medical decision makers and with regard to the domain-specific knowledge, understanding, and skills to be acquired during medical training are often debated (Kelly et al., 2015). Previous research describes several personal variables, such as confidence and prior knowledge, which influence the processing and acquisition of domain-specific knowledge (Posner et al., 1982; Cordova et al., 2014). While medical students themselves recognize the importance of science-based decision-making, they often do not feel confident in their ability to do so (Pruskil et al., 2009) although there is also evidence of overconfidence effects (Borracci and Arribalzaga, 2018). This, in turn, may be due to deficits in their ability to evaluate their own knowledge and recognize deficiencies therein (Kruger and Dunning, 1999; Eva et al., 2004). With regard to the learning progressions of medical students, making professional decisions and solving problems in clinical contexts require not only the acquisition of knowledge but also an increase in confidence in their medical knowledge (Khan et al., 2001). However, in multiple-choice (MC) tests, which are commonly used to assess knowledge in medical studies as well as in the written final examinations that preclinical and clinical medical students have to pass, confidence in one’s own decisions is not (routinely) assessed. Thus, participants may give correct answers despite having low confidence in their knowledge or by simply guessing (Walstad et al., 2018; Roeper et al., 2020), which may indicate that they are not ready to assume the responsibilities of a medical profession.

Physiology is one of the most basic topics in medical higher education and the basis for all applied fields of medicine. Nathial (2020, p. 2) describes anatomy and physiology as medical fields associated with the structures (e.g., the structure of red blood cells, related to anatomy) and functions of the human body; physiology describes and investigates the functions and cooperation of the different anatomical structures (e.g., transport of oxygen *via* red blood cells, Nathial, 2020, p. 2; for another example, see Section “Test Instruments”). The particular complexity of physiological knowledge is mainly caused by many interrelated levels that interact dynamically with each other (Hmelo-Silver et al., 2007). An understanding of anatomy and physiology is, therefore, seen as fundamental for working in medicine (Nathial, 2020, p. 2). There is evidence that experts and novices differ mainly in their understanding of causal behaviors and functions and less in their knowledge of structures (Hmelo-Silver et al., 2007). Consequently, it is a matter of learning about complex systems (Burggren and Monticino, 2005), which—as Hmelo-Silver and Azevedo (2006) argue—is difficult for various reasons: the sheer volume of relevant topics, the lack of direct experience of these concepts, the violation of our intuitive assumptions and preconceptions, and thus requires cognitive but also metacognitive and affective resources.

The differences between anatomy and physiology are not only reflected in their degree of difficulty of understanding but also result in difficulties in measuring the understanding of physiological functions. In this study, it is not possible to simply ask for the names of certain structures; rather, e.g., the use of MC tests requires particularly careful development of adequate distractors that also address typical misconceptions (Roeper et al., 2020). Taking into account the specific features of the field of human physiology (functions and complex interrelationships and preconceptions), this results in a particularly challenging valid and reliable assessment of students’ knowledge levels.

Especially in German-speaking countries, numerous studies have shown that difficulties in understanding and misconceptions are prevalent in this content area. Physiology is an important part of the first stage of studies and the first of two nationwide exams in Germany, with an average of 11.5% of students failing the most recent exam in autumn 2020. In this exam during autumn 2020, for example, the physiology section of the test was the part with the lowest average solution frequency (e.g., IMPP, 2020). As an example of a misconception, due to pre-academic conceptions (beliefs) that the brain is some kind of “computer,” medical students often fail to see the fundamental flaw of this metaphor and consequently struggle to understand brain functions correctly. While software and hardware can be easily distinguished in computers, brains function as hybrids (wetware), with software (physiology) constantly altering (plasticity) hardware (anatomy). This misconception is a significant roadblock in understanding how brains function in a healthy or diseased state.

Based on the existing research on “confidence testing” and “knowledge assessment” (refer to Section “Conceptual-theoretical Background and Research Hypotheses”), the issues of (i) how preclinical medical students acquire knowledge in physiology over their course of study and simultaneously gain confidence in the correctness of their knowledge as well as (ii) the interrelations between these processes, and (iii) how they affect the *development* of domain-specific knowledge are investigated in this pre–post study. More specifically, we empirically identified certain learning profiles depending on the combination of students’ knowledge and confidence levels at both measurements, which significantly differ in terms of the considered personal characteristics (e.g., prior education). Our findings provide some insights into this complex and reciprocal relationship between knowledge development and confidence, which can lead to first suggestions on how to train preclinical medicine students to reflect on their reasoning and to initial ideas on how to improve their decision-making and problem-solving skills.

## Conceptual-Theoretical Background and Research Hypotheses

The acquisition of professional knowledge and skills is the central goal of higher education. Miller (1990) developed a pyramid model focusing on the basics of medical actions, with factual knowledge serving as the basis for practical knowledge and the performance of medical actions. Patel et al. (1999) distinguish between the two basic types of knowledge, which are also used in the medical field: the verbalized knowledge of facts and concepts, which are learned explicitly, and more implicit, procedural knowledge, which underlies heuristics and tends to be acquired through practical experience in hospitals or similar settings. In the following, we briefly refer to the theoretical considerations that could underlie knowledge and confidence.

Although domain-specific knowledge is of critical importance for understanding the scientific rationales of practical work in medicine (Wijnen-Meijer et al., 2013a,b), a recent meta-study (Cate et al., 2018) indicated that the issue of competence development and knowledge acquisition in medical education has not been systematically addressed in research so far. While medical education relies on a variety of admission tests (e.g., Spiel and Schober, 2018) as well as multimedia-based performance assessments, research still suggests a substantial deficit of studies that measure students’ domain-specific knowledge acquisition throughout the medical school with valid and reliable test instruments (Cox and Irby, 2007; Prediger et al., 2020).

A majority of recent studies have focused on more general competence facets such as medical problem-solving and clinical reasoning (e.g., Custers, 2018). This research is often based on the “dual process” theory (heuristic and analytic processes; Evans, 1984; refer also to Chaiken (1980) heuristic systematic model), which particularly emphasizes the role of “intuitive cognition” (Patterson and Eggleston, 2017). When solving a domain-specific task and, for example, choosing among several response options, this kind of reasoning can be associated with Kahneman (2011) “System 2,” i.e., a “subjective feeling of confidence” (Sanders et al., 2016). In this context, some studies investigated to what extent students have confidence in their acquired knowledge or, in contrast, base their domain-specific decision-making or problem-solving on “strategically selected options” (e.g., straight-out guessing rather than using their knowledge; Weier et al., 2017; Lubarsky et al., 2018). More specifically, Gardner-Medwin (1995) considers not only the factual correctness of an answer but also the participants’ confidence in their answers to be central sources of information when analyzing knowledge acquisition/development. Overall, this research indicates that the awareness of confidence levels in relation to levels of domain-specific knowledge is important in domain-specific learning and expertise development.

According to the “cognitive continuum theory” (CCT), (i.e., an extension of the dual process model, Hammond et al., 1987; Hammond, 1996), when working on a task, solving and decision-making processes can be based on prior knowledge and/or heuristic approaches and subjective “good feelings.” Based on this framework, it can be assumed that a wrong item response can also be the result of incorrect knowledge or a misconception or even an “intuition-based” decision that might also be exhibited, e.g., by guessing on individual items (refer to for modeling guessing effects in MC tests, e.g., Walstad et al., 2018). For instance, in coglabs studies (Brückner, 2017) with retrospective think-aloud interviews, students often reported making a decision in favor of a certain response option because of their “good feeling.” This can be clearly distinguished from guessing because of a lack of relevant knowledge or the selection of an incorrect answer because of a misconception. Based on these studies, it can be argued that an incorrect item response can also result from a misconception presented in an individual distractor.

Based on research on competence testing (e.g., Bruno, 1993; Davies, 2006), confidence in the correctness of one’s knowledge, and (domain-specific) task-related confidence, can be considered as an appropriate indicator of the extent to which a student’s response is based on knowledge vs. strategic guessing. Confidence testing is based on the assumption that the level of confidence in the response to an item in a knowledge test can be used to more precisely categorize individuals according to their abilities, indicating a closer reciprocal link between knowledge level and confidence (Kolbitsch et al., 2008). A recent meta-study indicates (Stankov, 2013), besides individual variables such as self-concept and self-efficacy, that confidence is found to be one of the best predictors of academic achievement (measured using domain-specific knowledge tests), with the highest predictive validity compared to the other “self-beliefs.” This research indicates a major influence of confidence on the development of knowledge.

In the context of conceptual change, this relationship was examined in a pre–post study by Cordova et al. (2014): Based on students’ prior knowledge and confidence in their knowledge (and also considering further characteristics such as self-efficacy and interest), three empirically distinct learning profiles (“low”: low confidence and prior knowledge; “high”: high confidence and prior knowledge; and “mixed”: high confidence and low prior knowledge) were identified, which may significantly impact the conceptual learning of college students. This is further supported by the findings of Alexander and Murphy (1998), who also identified three distinct student profiles, and changes in these profiles over an academic term as well as their impact on domain learning (Alexander et al., 1997).

Based on a few existing studies with a particular focus on medical students (e.g., Khan et al., 2001; Fitzgerald et al., 2003), both a relationship between students’ performance in knowledge tests and their confidence in their response as well as the development of this relationship over the course of studies can be expected. Since the available findings are partly non-conclusive, further empirical investigations are required to analyze the interaction of confidence and learning and decision-making of medical students in preclinical courses. As some studies indicate that confidence in one’s erroneous knowledge, e.g., due to a misconception, can also increase throughout a course of study (Fitzgerald et al., 2003; Hall et al., 2007), more longitudinal research on the development of knowledge and confidence is required.

In this context, Khan et al. (2001) studied the relationship between the correctness of knowledge, the confidence in correct knowledge, and the use of correct knowledge in decision-making: “By ignoring this aspect of learning, when a student correctly responds to a question, it is not possible to determine whether the correctly identified knowledge is usable for decision making or not” (pp.160–161). The research group emphasizes that plain ignorance or misinformation can be responsible for an incorrect answer (or incorrect knowledge), whereby they see misinformation as even worse: “Misinformation is particularly dangerous because the student strongly believes that the wrong answer is correct” (pp.160–161). This finding indicates the particular relevance of the level of students’ confidence in the correctness of their knowledge for learning in a medical study domain and for uncovering possible misconceptions.

Building on the existing theoretical and methodological framework, we defined the following five hypotheses *(H)* to be tested in this study.

With regard particularly to the theory of domain learning (Alexander et al., 1995, 2018) as well as the curricular validity of the knowledge test used in this study, which comprises the contents and concepts taught in the physiology lecture and seminar series, we assume that the 1st year students have only little relevant previous knowledge from high school and vocational medical training at t1 and that the level of their knowledge increases over the course of the semester, manifesting in higher test scores at t2.

H1: The students’ level of domain-specific knowledge is significantly lower before (t1) than after actively participating in the physiology seminar series (t2).

Recent research on knowledge acquisition and its determinants suggested several individual student characteristics that may significantly contribute to differences in students’ knowledge levels and their development. Cognitive variables, such as general *cognitive ability* (Brandt et al., 2019) and *prior knowledge* (Shing and Brod, 2016), as well as psychological variables, such as *motivational factors* (Rotgans and Schmidt, 2017), *personal characteristics* (as, e.g., gender, e.g., Haq et al., 2005; Firth-Cozens, 2008), and *confidence* (Rudolph et al., 2017), were repeatedly identified as significant predictors. Therefore, as *H2*, we assume that these personal characteristics significantly contribute to explaining students’ domain-specific knowledge levels at t1 and t2 and their development (difference score), as:

H2a: Indicators of prior knowledge [advanced school courses or vocational training) predict students’ knowledge levels.

H2b: Indicators of general cognitive ability (intelligence test score and grade of university entrance qualification (UEQ)] predict students’ knowledge levels.

H2c: (Intrinsic) learning motivation predicts students’ knowledge levels.

H2d: Task-related confidence predicts students’ knowledge levels.

H2e: Socio-biographical characteristics, e.g., gender, predict knowledge levels.

In particular, in many studies, female students show both a significantly lower degree of knowledge and confidence in MC tests (Parker, 2006; Owen, 2012), which was also reflected in a different response behavior than male students (e.g., more missing values; Walstad et al., 2018).

Task-related confidence has already been determined as a significant influencing factor for knowledge test values (Parker, 2006; Kleitman et al., 2012). Based on these studies, we further assume that task-related confidence increases with increasing knowledge levels.

H3: The average level of students’ confidence in their (correct as well as incorrect) responses is higher at t2 than at t1.

In addition, with reference to the reported findings, we expect that confidence is also influenced by personal characteristics (e.g., for prior knowledge, refer to Dinsmore and Parkinson, 2013), leading to H4,

H4: Personal characteristics significantly contribute to the explanation of students’ confidence levels at t1 and t2 and their development (difference score).

Therefore, the assumed relationships in *H4a–e* are formulated and tested empirically using the same strategies employed in *H2a–e* (for details, refer to Section “Statistical Methods”).

Alexander (2013a) emphasize the importance of examining not only the participants’ confidence in their responses but also and especially the issue of calibration, i.e., the relationship between their self-estimation and performance (for deeper insights, refer to Alexander, 2013b). As existing studies indicate a mutual influence between knowledge and confidence as well as corresponding learning profiles, we assume in *H5* that:

H5a: Students’ domain-specific knowledge is positively related to their confidence when answering the test questions both at t1 and t2 and between the difference scores (t2 – t1) in both knowledge and confidence.

H5b: On the basis of correlations between the change in domain-specific knowledge levels and task-related confidence, students can be empirically categorized into three groups, with specific personal attributes (such as learning motivation, general cognitive ability, and prior education) that characterize these groups.

## Materials and Methods

### Study Design

The analyses were based on the data from a study supported by the Rhine-Main-Universities (RMU) fund, in which overall 169 students of medicine who actively participated in a 2nd semester seminar series on physiology at a university in the Rhine-Main region, Germany, were tested at the beginning (t1) and end (t2) of the seminar series (2019 summer term to 2019/2020 winter term). In total, 137 students participated at t1, 135 at t2. Out of these, 97 students participated in both measurements, whereby this longitudinal sample forms the basis of the analyses presented in this study. The surveys took place at the beginning of the mandatory seminars and under controlled test conditions in a group setting. For this purpose, test administrators were given specific training. According to the university’s curriculum, the seminar series is intended for the 2nd to 3rd semester medical students. It was not possible to implement a comparable control group in the field survey as all students at this stage of medical studies are required to participate in the seminars (for limitations, refer to Section **“Limitations”**). Though participation in the study was voluntary, all students who attended the seminar series took part. As an incentive for test motivation, the students were given an opportunity to view their test results (on sum score level, not item level) online after both measurements and to obtain individual feedback on their knowledge development^1^.

### Test Instruments

Paper–pencil questionnaires were used at both measurements. In t1, in addition to their domain-specific knowledge in physiology, participants’ socio-demographic data such as gender, native language, and previous education as well as further information such as general cognitive ability were assessed. In t2, the identical knowledge test was used again, and further characteristics relevant to the learning process, including motivation, were assessed. Completing the survey took on average 30 in t1 and 25 min in t2.

#### Physiology Knowledge Test in t1 and t2

Knowledge of physiology was assessed using 12 newly developed single-choice items with five answer options each, a test format that students are familiar with from their medical studies (an example item: “How is water predominantly transported from the extracellular space through cell membranes? (A) Active transport *via* solvent drag, (B) carrier-mediated symport with chloride, (C) Primary active transport, (D) along an osmotic gradient *via* connexins, and (E) along osmotic gradients *via* aquaporins.” The single-choice items focus on assessing primarily (declarative) knowledge about basic concepts of physiology. Due to the design of the distractors, however, a deeper understanding of the content is required to select the correct answer from the given answer options^2^ (for further details on the assessment approach underlying the test, see Roeper et al., 2020).

To prevent the students from cheating on the test, two versions of the questionnaire were created, with the same questions but in reverse order. The same test was used at t1 and t2. Correct answers were scored with one point, while missing answers (as an indicator of non-knowledge, Baker and Kim, 2004) and incorrect answers were each scored with 0 points, i.e., a maximum of 12 points could be achieved. Dichotomous coding is a strict variant of scoring that is commonly used for MC items (e.g., Kulhavy and Anderson, 1972; Andrich and Kreiner, 2010; Lee et al., 2011; Durning et al., 2015). A total score was calculated for each test taker from the 12 responses. Due to the limited test time in this field study, a relatively small number of items was used. To cover as many basic physiological concepts as possible, the items covered a variety of topics. This led to low reliability of 0.511 (for limitations, refer to Section “Limitations”). To determine the construct validity of the test, we conducted a confirmatory factor analysis (CFA). The CFA results are consistent with the assumption of the test developers that the 12 test items cover a comprehensive construct with many facets, which are all based on one common latent factor, i.e., knowledge of physiology. The model with an assumed one-dimensional solution shows a satisfactory fit with respect to most CFA fit indices at both measurements (t1 and t2); only the standardized root mean residual (SRMR) is not optimal (Table 1). Exploratory factor analyses also indicate the one-dimensional model. With regard to the order of the questions in the test questionnaires, no significant difference in the total score could be determined at both measurements (t1: *p* = 0.573 and t2: *p* = 0.847). There are also no significant differences in the item difficulties of the 12 items between t1 and t2 (χ^2^ < 0.01 – χ^2^ = 2.29, *p* = 0.131 – *p* = 0.993).

**TABLE 1:** Fit indices of confirmatory factor analysis (CFA) with a single-factor solution.

Model	*X* ^2^	*df*	*X^2^/df*	RMSEA	CFI	SRMR
t1	50.777	54	0.940	< 0.001	1.000	0.138
t2	53.701	54	0.994	< 0.001	1.000	0.124

#### Confidence in Responses to the Domain-Specific Test Items

To measure task-related confidence, after each task of the domain-specific test, a 4-level Likert scale was used to ask participants to what extent they were certain that their solution was correct (exact wording (translated): To what percentage are you convinced that the answer you have given is correct?). The test participants had to choose one of the four options: 0 - 25 - 75 - 100%. A mean value was calculated for all items at both measurements and used as a sum score. With a Cronbach’s α of 0.775, the internal reliability of the construct “confidence” can be considered acceptable.

#### Indicators of General Cognitive Ability

Two variables were used as indicators of general cognitive ability. As a common and easily measured indicator, the average grade of students’ UEQ was recorded. In addition, to indicate general cognitive ability, the scale “Choosing figures” of the Intelligence Structure Test (IST-2000 R, Liepmann et al., 2007) was used as an objective measure in the questionnaire. Due to the assumed stability of this construct (and the restricted test time), intelligence was only assessed at t1. The IST comprised 20 single-choice items for figural reasoning, whereby students had to work out which of the five given figures could be created by piecing together ten fragments. The test time was limited to 7 min. Correct responses were scored with one point, while missing answers (as an indicator of non-knowledge) and incorrect responses were each scored with zero points so that a maximum of 20 points could be achieved. A sum score was calculated for statistical analysis. The fit indices of the CFA indicated that the model with an assumed single factor solution fits the measured data satisfactorily regarding nearly all fit indices (root mean square error of approximation (RMSEA) = 0.041, weighted root mean square residual (WRMR) = 0.958, comparative fit index (CFI) = 0.784).

#### Previous Learning Opportunities

With regard to pre-university learning opportunities, the participants were asked whether they had completed advanced courses for several relevant subjects in high school (in biology, chemistry, physics, and mathematics; (t1); in this study, multiple answers were possible. The participants were also asked about the completion of any medical vocational training (t2).

#### Learning Motivation

Intrinsic learning motivation was measured with a short scale (adapted from the scale Vallerand et al., 1992; Schiefele et al., 1993), which consisted of four items (e.g., “I study for my (degree) course because I enjoy working with the content”) and could be answered on a 6-point scale from 1 “applies fully” to 6 “does not apply at all” resulting in a score with an inverted scale, where lower scores signify higher motivation. The reliability analyses showed a Cronbach‘s α of 0.833. The fit indices of the CFA show that a one-dimensional model is quite suitable for the measured data (e.g., RMSEA = 0.087, SRMR = 0.023, CFI = 0.990).

### Sample Description

The longitudinal sample and the subsamples t1 and t2 are described in Table 2.

**TABLE 2:** Descriptive statistics for the samples at t1, t2, and for the matched sample.

Variables	t1 *N* = 134	t2 *N* = 132	Match *N* = 97
Gender, male, n (%)	37 (27.61%)	40 (30.30%)	29 (29.90%)
Preferred communication language, German, n (%)	116 (86.57%)	n/a	87 (89.69%)
Age, mean ± *SD*	22.01 ± 3.642	22.01 ± 3.642	22.01 ± 3.897
UEQ grade^1^, mean ± *SD*	n/a	1.45 ± 0.541	1.44 ± 0.513
IST sum score, mean ± *SD*	12.46 ± 3.46	n/a	12.91 ± 3.324
learning motivation^2^, mean ± *SD*	n/a	2.08 ± 0.683	2.11 ± 0.684
Educational background:			
Advanced sciences course at school, n (%)			
None	27 (40.70%)	30 (22.73%)	17 (27.87%)
Biology	35 (40.70%)	70 (53.03%)	26 (42.62%)
Chemistry	2 (2.33%)	32 (24.24%)	2 (3.28%)
Physics	1 (1.16%)	15 (11.36%)	1 (1.64%)
Mathematics	17 (19.77%)	62 (46.97%)	11 (18.03%)
More than one course	4 (4.65%)	n/a	4 (6.56%)
Medicine-related vocational training, n (%)	n/a	16 (12.12%)	12 (12.37%)

*^1^UEQ = university entrance qualification. In Germany, lower numbers indicate better grades (1–6).*

*^2^ Inverted scale, lower numbers indicate higher motivation scores.*

### Statistical Methods

The matching of the longitudinal data was performed using R, packages *dplyr* and *haven* (R Core Team, 2018), based on a pseudonymized, unchangeable six-character code (e.g., including the second letter of the mother’s first name) generated by the test takers. In addition to descriptive and factor-analytical analyses to investigate the internal test structure and its dimensionality (Cronbach‘s α; exploratory and CFA), regression analyses were also conducted to investigate the research hypotheses (*H*) *2* and *4*. In addition to the indicators described in Section “Test Instruments,” the regression analyses always include language, age, and gender as control variables. In addition, differences in mean values were tested for significance using *t*-tests and ANOVAs (*H1* and *H3*). Due to a relatively small sample and the correspondingly small subsamples, the effect sizes were also reported in addition to the significance levels (Cohen’s *d* for two groups, Omega^2^ [ω^2^] for more than two groups, and Cramer’s *V* for frequency distributions; refer to Cohen, 1988; Ellis, 2010; Grissom and Kim, 2012; Field, 2013).

The measurement invariance analyses for the construct “knowledge” conducted at t1 and t2 indicate a scalar invariance (WLSMV estimator for categorical variables, e.g., RMSEA = 0.036, WRMR = 1.042), whereas a metric invariance between t1 and t2 can be determined for the construct “confidence” (MLMV estimator for continuous variables, e.g., RMSEA = 0.041, SRMR = 0.093, CFI = 0.802).

Regarding *H5a*, based on the students’ confidence levels and the correctness of the item responses, in the first step, four different combinations of these variables were distinguished. Therefore, the confidence items were split into low and high, with “low” being 0% or 25% and “high” being 75% or 100%.

To test *H5b*, latent class analyses (LCAs) were calculated in the following step, indicating a model with a three-factor solution according to the Akaike information criterion (*AIC*) and Bayesian information criterion (*BIC*) fit indices (refer to Section “Results”). The fit values for the three- and the two-factor model are fairly similar. The three-factor model was chosen, which is also in line with prior research (e.g., Cordova et al., 2014).

The analyses were performed using Stata 15 (Stata Corp, 2017), and MPlus Version 7 was used for the latent analyses (Muthén and Muthén, 1998-2011). The application prerequisites for the applied methods were also checked and confirmed.

## Results

### Level of Knowledge in Physiology Before (t1) and After (t2) Attending the Seminar Series *(H1)*

Regarding *H1*, students’ levels of domain-specific knowledge before (t1) and after (t2) attending the physiology seminar series were examined. As expected, the knowledge score differs significantly between the two measurements (*t*(93) = −16.211, *p* < 0.0001, Δ3.45, *corr* = 0.380, Cohen‘s *d* = 2.07): While at t1 the students had an average score of 4.39 (± 1.497), the average score at t2 was 7.84 (± 2.096) indicating a significant increase in knowledge after participating in the seminar series.

### Influencing Factors on Students’ Knowledge Level and Its Development *(H2)*

When analyzing the students’ domain-specific knowledge level at t2 as a dependent variable (*H2*), regression analyses showed that confidence in the given (correct or incorrect) response at t2 (β = 0.554, *p* < 0.001) was the most significant predictor in this model (*H2d*). Learning motivation was also a significant predictor of domain-specific knowledge level at t2 (*H2c*, β = −0.200, *p* = 0.026), whereas the two indicators of general cognitive ability (*H2b*) and gender (*H2e*) were not. In addition, the knowledge score at t1 (β = 0.199, *p* = 0.024) was less predictive than the level of confidence at t2. Among the assessed indicators for prior knowledge, attendance of advanced courses in biology, math, and physics in high school as well as students’ socio-biographical characteristics such as gender did not contribute to the prediction *(H2a)*. Overall, the regression model achieved an *R*^2^ of 50.96% (adj. *R*^2^ = 40.60%).

When analyzing the difference score between the sum scores in physiology knowledge at t1 and t2 as a dependent variable (*R*^2^ = 30.58%, adj. *R*^2^ = 17.09%), the significance of the predictor confidence (in correct as well as incorrect responses) at t2 (β = 0.414, *p* = 0.001) and the predictor learning motivation (β = −0.235, *p* = 0.031; inverted scale) was confirmed. Even when confidence was included in the model as a difference score from t1 to t2, it showed a statistically significant effect (β = 0.235, *p* = 0.038), while the indicators of prior education and gender were not significant.

### Level of Confidence in Their Test Response Before (t1) and After (t2) Attending the Seminar Series *(H3)*

There was a positive development from an average confidence level of 1.92 (± 0.404) at t1 to an average level of 2.74 (± 0.452) at t2. This difference also became significant with a strong effect size (*t*(86) = −16.867, *p* < 0.001, Δ0.82, *r* = 0.454, Cohen‘s *d* = 1.94).

### Influencing Factors on Students’ Confidence in Their Test Response *(H4)*

When analyzing the determinants of confidence at t2 (*H4*), the regression model, taking into account the knowledge scores, socio-demographic characteristics, indicators of general cognitive ability, and indicators of prior knowledge, shows that confidence at t1 was a significant predictor (β = 0.351, *p* < 0.001). More significant than confidence at t1 was the knowledge score at t2 with a β of 0.537 (*p* < 0.001; *H4d*). Besides gender (β = 0.193, *p* < 0.033; *H4e*), other covariates were not significant (*H4a–c*). However, this regression model already achieved an *R*^2^ of 56% (adj. *R*^2^ = 46.75%).

If the development of confidence was focused as a difference score, the knowledge score at t2 remained the only significant predictor (β = 0.434, *p* < 0.001, *R*^2^ = 32.10%, adj. *R*^2^ = 18.90%).

### Relation Between Students’ Domain-Specific Knowledge (Changes) and Their Confidence at t1 and t2 *(H5a)*

When analyzing and comparing the correlations between the mean confidence level and the sum score in the knowledge test at t1 and t2, it became evident that at t1 the correlation of *r* = −0.039 was not significant, while it was significantly higher at t2 with a correlation of *r* = 0.529. At *r* = 0.208, the change in the confidence level was also related to the change in domain-specific knowledge. This is in line with the results of the regression analyses for *H2*, which showed that the confidence level at t2 was of particular importance, while the confidence level at t1 was negligible (β = 0.191, *p* = 0.070). Similar results were also found for the difference score of the knowledge test as a dependent variable (confidence level t1: β = 0.038, *p* = 0.754).

Furthermore, based on the students’ confidence levels and the correctness of the item responses in each measurement point, four combinations were distinguished: (1) *confident and correct*, (2) *not confident and correct*, (3) *not confident and incorrect*, and (4) *confident and incorrect*. The average proportions of the four combinations at t1 and t2 are shown in Table 3.

**TABLE 3:** Average proportions (in %) of the four combinations at t1 and t2.

Case	t1	t2	Δ	*p*
confident and correct	13.08	45.83	+ 32.75	< 0.001
confident and incorrect	9.92	11.83	+1.91	0.395
not confident and correct	24.08	19.00	–5.08	0.014
not confident and incorrect	52.92	22.50	–30.42	< 0.001

*t-tests were used for significance testing.*

Overall, the pre–post test data show that the number of *confident and correct* cases increased on average from t1 to t2 (*x*^*t*1^ = 1.58 ± SD^*t*1^ = 1.009, *x*^*t*2^ = 5.56 ± SD^*t*2^ = 2.641, *t*(88) = −15.680, *p* < 0.001*, *d* = 3.68) and that the number of *not confident and incorrect* cases decreased on average from t1 to t2 (*x*^*t*1^ = 6.33 ± SD^*t*1^ = 1.700, *x*^*t*2^ = 2.68 ± SD^*t*2^ = 1.921, *t*(87) = 15.355, *p* < 0.001*, *d* = 1.75). With regard to the *not confident and correct* cases, a slight decrease became evident between t1 and t2 (*x*^*t*1^ = 2.85 ± SD^*t*1^ = 1.614, *x*^*t*2^ = 2.315 ± SD^*t*2^ = 1.669, *t*(88) = 2.508, *p* = 0.014*, *d* = 0.268). However, no significant difference between t1 and t2 can be determined with regard to the *confident and incorrect* cases (*x*^*t*1^ = 1.22 ± SD^*t*1^ = 1.178, *x*^*t*2^ = 1.36 ± SD^*t*2^ = 1.323, *t*(87) = −0.854, *p* = 0.395, *d* = 0.09).

### Profiles Based on Confidence and Knowledge (Change) and Specific Characteristics (*H5b*)

As the inferential statistical analyses showed significant differences in students’ response behavior regarding confidence (in their correct as well as incorrect responses) and performance in the knowledge test at t1 and t2, an LCA was conducted to identify further possible correlations and differences within the groups depending on the confidence level.

First, an LCA was conducted on the basis of confidence at t1. As mentioned above, the three-class solution was chosen for confidence at t1 based on the LCA model parameters *AIC* and *BIC* (t1: three-class solution with *AIC* = 133.398, *BIC* = 150.368; Van Den Bergh and Vermunt, 2019). The LCA indicated three profiles: a low-confidence group (24.26%), a medium confidence group (42.01%), and a high-confidence group (33.73%). Supplementary Table 1 shows which variables differ among the three groups at t1. While there was a difference between the groups in terms of confidence levels at t2, there was no significant difference in the knowledge scores at t1 and t2. At a marginal level of significance, there is a difference in terms of intelligence test scores in favor of the low-confidence group, which was not consistent with the User Experience Questionnaire (UEQ) grade. When comparing the groups, it became evident that a higher proportion of the medium confidence group completed medical vocational training, while members of the group with the highest confidence at t1 were significantly more likely to have taken an advanced physics course in high school.

An LCA also determined a three-cluster solution for the confidence level at t2 with the following groups (t2: three-class solution with *AIC* = 119.193, *BIC* = 134.124): low confidence (5.92%), medium confidence (21.89%), and high confidence (72.19%). The clusters for confidence at t1 and t2 show only a weak correlation of *r* = 0.179. Supplementary Table 2 shows the differences among the three groups at t2. The students from the high-confidence group at t2 had a high level of confidence at t1 as well, while their knowledge level at t1 was not notably higher than that of other groups; and showed the highest increase in knowledge between t1 and t2.

Next, a *combined* latent cluster that best describes the change in knowledge and confidence from t1 to t2 was also determined. Similar to t1 and t2, the most appropriate cluster solution according to the model fit indices (three-class solution with *AIC* = 532.703, *BIC* = 558.450) resulted again in three groups: *knowledge gainers* (7.69%), *confidence gainers* (11.24%), and *overall gainers* (81.07%). Table 4 shows the differences in the included covariates. When analyzing the difference scores for confidence and knowledge, the expected differences between the two groups became apparent, indicating a higher growth in both confidence and knowledge among the group of overall gainers. This group also has the highest confidence level at t2 and the highest knowledge level at t2, while the confidence gainers have the highest knowledge level at t1. The latter group also has the highest proportion of students who have completed medical vocational training (only marginally significant).

**TABLE 4:** Differences between the three profiles regarding changes in confidence and knowledge.

	knowledge gainers	confidence gainers	overall gainers		Tukey post-estimation			
				
	*M* ± *SD* (n)/%	*M* ± *SD* (n)/%	*M* ± *SD* (n)/%	*F/*χ*^2^, p*	*p* low vs. medium	*p* medium vs. high	*p* low vs. high	ω^2^/Cramer‘s *V*
Confidence level at t1	2.14 ± 0.351 (13)	1.92 ± 0.383 (18)	1.96 ± 0.411 (94)	1.37, 0.258	0.278	0.911	0.281	0.006
Confidence level at t2	2.19 ± 0.394 (13)	2.64 ± 0.369 (18)	2.83 ± 0.439 (93)	13.68, <0.001	0.010	0.200	<0.001	0.170
Knowledge test score at t1	3.38 ± 0.768 (13)	4.84 ± 1.675 (19)	4.26 ± 1.622 (102)	3.32, 0.039	0.030	0.308	0.142	0.033
Knowledge test score at t2	6.38 ± 1.502 (13)	5.53 ± 1.50 (19)	8.30 ± 1.850 (100)	23.50, <0.001	0.374	<0.001	0.001	0.254
Confidence difference score	0.45 ± 0.235 (13)	0.67 ± 0.264 (17)	1.03 ± 0.306 (60)	13.68. <0.001	<0.001	< 0.001	<0.001	0.584
Knowledge test difference score	3.00 ± 1.732 (13)	0.68 ± 0.885 (19)	4.35 ± 1.556 (65)	46.11, <0.001	<0.001	<0.001	0.009	0.482
Learning motivation	2.13 ± 0.658 (13)	2.25 ± 0.635 (19)	2.035 ± 0.695 (100)	0.84, 0.433	0.886	0.423	0.874	−0.002
Intelligence test score	12.54 ± 3.688 (13)	11.68 ± 3.250 (19)	12.60 ± 3.48 (102)	0.56, 0.574	0.774	0.545	0.998	−0.007
UEQ grade	1.68 ± 0.650 (13)	1.35 ± 0.475 (19)	1.44 ± 0.534 (99)	1.59, 0.207	0.194	0.770	0.277	0.009
Age	22.92 ± 4.051 (13)	21.58 ± 2.694 (19)	21.97 ± 3.757 (98)	0.54, 0.582	0.565	0.905	0.651	−0.007
Sex, male	23.08%	31.58%	27.45%	0.285, 0.867	−	−	−	0.046
Language, German	15.38%	10.53%	13.73%	0.188, 0.910	−	−	−	0.038
Medical vocational training, yes	69.23%	94.74%	89.00%	5.201, 0.074	−	−	−	0.199
Advanced course in school in biology, yes	69.23%	47.37%	55.00%	0.647,0.724	−	−	−	0.070
Advanced course in school in chemistry, yes	15.38%	26.32%	25.00%	0.631, 0.729	−	−	−	0.069
Advanced course in school in physics, yes	0.00%	15.79%	12.00%	2.076, 0.354	−	−	−	0.125
Advanced course in school in math, yes	53.85%	42.11%	47.00%	0.427, 0.808	−	−	−	0.057
Advanced course in school in sciences, no	15.38%	26.32%	23.00%	0.543, 0.762	−	−	−	0.064

*^1^UEQ = university entrance qualification. In Germany, lower numbers indicate better grades (1–6). ^2^Inverted scale, lower numbers indicate a higher motivation score ω^2^ < 0.01 = very small, 0.01–0.06 = small, 0.06–0.14 = medium, and > 0.14 = large. Cramer‘s V < 0.1 = negligible, 0.1–0.29 = small, 0.3–0.49 = medium, and ≥ 0.5 = large effect.*

## Discussion

### Summary and Conclusion

In a pre–post study, the medical students’ knowledge development in physiology was assessed. We found a significant increase in the students’ knowledge in physiology from t1 to t2 with large effect size, supporting *H1*. Task-related confidence at t2 (also when controlling for other personal covariates such as intelligence, learning motivation, and gender) was revealed to be the strongest predictor of the knowledge score at t2 and the increase in knowledge, supporting *H2d*. Learning motivation was also a significant predictor, supporting *H2c*. Overall, 51% of the variance of the knowledge score from t2 can be explained, also with regard to the difference score, even though the explained proportion of variance was 31% for the latter. The lack of predictive power of prior learning opportunities (*H2a*) may possibly be due to the relatively narrow conceptualization and empirical operationalization of “prior knowledge” in this study. For instance, current research provides further insights into the understanding of “prior knowledge” that should be considered in future work (McCarthy and McNamara, 2021). In addition, a current meta-analysis by Simonsmeier et al. (2021) shows the particular importance of analyzing the conditions under which prior knowledge has an effect on learning processes, which require further investigation.

As it is assumed that the students’ knowledge increases over their course of study in physiology, a significantly higher level of confidence at t2 was expected and confirmed by the data, with a large effect size (supporting *H3*). Remarkably, a high level of confidence at t1 was a significant predictor of a high level of confidence at t2, which indicated high stability and correlation of this variable. With respect to the difference between the two confidence scores at t1 and t2, only knowledge at t2 was a significant predictor in the model explaining confidence, supporting *H4a*.

The results for *H5* indicate that at t1, confidence is less significant for predicting knowledge, while at t2, confidence is a much more significant predictor of knowledge and knowledge change that may occur through actively participating in the physiology seminar series. This supports the abovementioned findings (Section “Conceptual-theoretical Background and Research Hypotheses”) that higher knowledge (at t2) may result in higher confidence (at t2) and vice versa. However, the students who are *confident but incorrect* constitute one particularly striking learning profile (i.e., *confidence gainers*) as the pre–post test results indicate no significant difference between the *incorrect responses at t1 and t2*. This finding indicates that among this group of students, incorrect knowledge or misconceptions might become established for certain domain-specific concepts captured in these test items. Identifying learning profiles of this kind enables better characterization of the possible misconceptions in medical education, and in turn, targeted interventions to correct them. Given that this group represents about 10% of the medical student population, teaching resources can now be efficiently focused on addressing potential misconceptions (refer to Section “Implications for Research and Practice”).

The emerging dissociation between confidence and knowledge might also be linked to the way medical curricula are commonly structured in Germany, where a 2-year preclinical phase focuses on the acquisition of the knowledge and skills needed to understand and utilize the basic science underpinning medicine (Fritze et al., 2017a,b). However, in preclinical subjects like physiology, this is mostly done using canonical textbooks, and students tend to focus on memorizing and paraphrasing a long and ever-growing list of facts from textbooks instead of learning to trust their own developing sense of scientific argumentation (i.e., flexible problem-solving with confidence).

At t2, the significance of the classification in terms of the knowledge score becomes evident, which is comparable to previous results. After attending a physiology seminar over one term, students appear to have built a subject-related knowledge base. Confidence is, therefore, also a meaningful indicator when explaining and predicting knowledge scores. However, the direction of the relationships between the two constructs knowledge and confidence, and their development between t1 and t2 remains unclear. Overall, there are higher proportions of students in the high-confidence group in the cluster at t2. The correlation between the confidence clusters t1 and t2 is not very high, which suggests that these clusters may change over the course of a seminar in physiology.

Mixed groups (LCA) of knowledge and confidence show fewer differences in terms of the covariates included. Confidence levels at t2 are the lowest in the group of *knowledge gainers*. Furthermore, the knowledge score at t2 differs significantly between the groups. The difference scores in knowledge and confidence, which were also the basis for generating and labeling this combined cluster, differ with high effects. Even if only at a marginally significant level, the group of *knowledge gainers* has the lowest proportion of students with completed vocational training. To further explain these learning profiles, more information in terms of student characteristics is needed.

When exploring the factors influencing both domain-specific knowledge and task-related confidence, as well as when considering the differences among the three clusters, intelligence—usually one of the strongest influencing factors according to other studies (e.g., Schwager et al., 2015; Wai et al., 2018)—explains only a small, non-significant amount of variance. One possible explanation may lie in the high pre-selectivity of the sample of medical undergraduates in terms of the cognitive study requirements, which leads to the high homogeneity of intellectual preconditions among this group. Indeed, the medical students scored substantially better on the IST test (*M* = 12.91 ± SD = 3.324) than a comparison group of students from business and economics (*M* = 6.57 ± SD = 1.766, *n* = 246; from another project, Zlatkin-Troitschanskaia et al., 2019a).

The gender effect on confidence was only weak and marginally significant. These results were in contrast with previous research (e.g., Walstad and Robson, 1997; Hambleton, 2005; Brückner et al., 2015) and might reflect specific features of the medical student population, which has become predominantly female. In the context of knowledge assessment and competence testing, this finding can be interpreted as an indicator of discriminate validity and test fairness and supports the implementation of assessments as presented in this study in medical education practice (Zlatkin-Troitschanskaia et al., 2019b).

### Limitations

The presented results should be interpreted in consideration of the limitations of this study. First, the sample size is relatively small (even though an entire student cohort was examined in the specific context of a physiology seminar series). Consequently, the subsamples (e.g., students who took an advanced course in chemistry at school) were also small, which might have caused sampling effects. Although this sample can be considered representative for medical students according to the German official statistics (in terms of the students’ main descriptive characteristics), it would be premature to draw general conclusions.

Second, the final sample of 97 students is limited as a basis for latent analysis and should also be considered cautiously with regard to the number of statistical analyses (for power determination, refer to Muthén and Muthén, 2002). To gain a comprehensive first insight into the relationship between confidence and knowledge development as well as into influencing factors, the multiple linear regression and LCA were carried out. Therefore, the results should be interpreted with caution and require testing in larger samples. Larger samples would also allow for more intensive parallelism checks (using item rasch theory (IRT) procedures), which were not carried out in this study.

Third, due to the nature of the field survey, and as all students at this stage of their medical studies had to attend the seminar series, it was not possible to establish a control group. This limits the interpretability of the causality of the identified effects. However, this is a general concern in higher education field research, since in field studies it is almost impossible to conduct a study with a control group. Therefore, the robustness of the results should be examined in similar follow-up studies with larger cohorts and more measurement points, and the study should also be conducted at other universities and in other countries.

Fourth, due to time restraints in this field study, only a short version of the knowledge test was used in the study, which reflected only a small part of both physiology curricula and the cognitive requirements in the preclinical phase. However, our aim with this short test was not to evaluate the teaching effects in medical education, but rather to examine medical students’ fundamental developmental tendencies and above all the relationships between confidence and knowledge, as demonstrated in the analyses presented in this study (refer to Sections “Relation between students’ domain-specific knowledge (changes) and their confidence at t1 and t2 (H5a)” and

“Groups based on confidence and knowledge (change) and specific characteristics (H5b)”). The low reliability of the test might be improved in the future by implementing polytomous scoring (e.g., Embretson and Reise, 2000); partial crediting for the short test is currently in progress and may be used for future improvement. Due to the low number of test items, it was not possible to design two parallel test versions, and carry-over effects cannot be fully ruled out by this study design. However, since there is an interval of about 6 months between t1 and t2, significant test-induced learning effects seem unlikely (e.g., Scharfen et al., 2018).

Fifth, the predictive validity of the assessed personal characteristics may be limited as short scales of general cognitive ability and learning motivation were used in this study. As some studies already suggest (e.g., Kruger and Dunning, 1999; Klymkowsky et al., 2006), the assessed “task-related” students’ confidence (students’ confidence in their responses) is not necessarily indicative of students’ self-confidence in their (metacognitive) abilities such as critically reflecting on their knowledge, problem-solving, and decision-making.

Despite these limitations, the examination of the relationship between knowledge and confidence as well as of the development of this relationship over time in a pre–post design contributes to the internal validity of the study results. A significant contribution to the still limited existing research in medical education as well as providing important insights into the seemingly reciprocal relationship between knowledge and confidence is made herewith (Section “Conceptual-theoretical Background and Research Hypotheses”). Based on prior research and the findings presented in this study; however, we cannot reach a satisfactory conclusion as to what particular (meta)cognitive and/or affective processes and trait- and/or state-like abilities underlie task-related confidence. Further, more differentiated research is needed.

### Implications for Research and Practice

Through a particular focus on the relationship between domain-specific knowledge and confidence dynamics, and the examination of the development of these variables in a longitudinal analysis, this study makes contributions to bridging current research gaps. Overall, with regard to the regression modeling of knowledge and task-related confidence at t2, we already explained more than 50% of the variance in students’ knowledge test scores and identified its most significant influencing factors. Compared to existing studies, which usually explain a relatively small amount of variance in students’ knowledge (refer to Section “Conceptual-theoretical Background and Research Hypotheses”), this large share of explained variance is particularly remarkable. This also indicates the high practical importance of the included influencing factors and, in particular, supports the claim that the valid assessment of confidence levels in relation to the levels of domain-specific knowledge and their development is important in domain-specific learning. At the same time, when looking at the still unexplained variance, additional research is required to explain the complex relationship between the development of (prior) knowledge and (task-related) confidence as well as the dynamics of this relationship in more detail.

To date, only little is known about the development of students’ confidence. In particular, the relationship between knowledge development and confidence requires more in-depth research. Moreover, the aspect of confidence should also be taken into account in formal knowledge assessments. Examining this relationship at the item level and, in particular, with a view to the item contents has the potential to contribute to a better understanding of how knowledge and confidence develop in relation to certain domain-specific concepts and/or types of tasks and problems. Therefore, future research should overcome the abovementioned limitations of the present study. In particular, in-depth (qualitative or mixed methods) analyses at the level of individual items and case studies of individual learning or development profiles have the potential to contribute to a more precise understanding of knowledge acquisition processes and their relation to confidence development.

This study offers initial insights; nevertheless, the chicken-or-egg dilemma remains: are students more confident because they have a higher level of knowledge, or do students choose the correct answers because they are more confident? More research on students’ mental processes, using think-aloud protocols and eye-tracking studies, is required to further investigate this reciprocal relationship.

Despite the limitations of this study, the results indicate that confidence is of particular importance and that a stronger focus should be placed on this aspect in education and training, especially in professions such as medicine, where fast (spontaneous) decision-making with confidence is essential. In this study, we argue that integrating the practice of asking students to critically reflect on their level of confidence in their task responses and their decision-making as early as during preclinical physiology courses is a useful exercise in several ways, for both students and instructors. For instance, in (self-)assessments, students will be trained to reflect on their reasoning and to improve their metacognitive ability to assess whether their confidence is justified. If combined with knowledge assessments, targeted interventions, and feedback (Butler et al., 2008), this will likely become an effective tool to increase student understanding and conceptual change. In addition to being a preparatory activity that will become more central (and complex) later on, when it comes to clinical decision-making, where multiple dimensions—including the needs and preferences of individual patients—need to be integrated, it also provides information about individual learning progressions in physiology. Confidence testing can provide teachers with valuable feedback about students’ learning difficulties, and identify certain content that students are uncertain about or areas in which they are misinformed (for deeper insights into current developments and perspectives in medical higher education, refer to e.g., Kopp et al., 2008; Blohm et al., 2015; Heitzmann et al., 2019).

*In summation*, a higher level of confidence in one’s own decisions can develop together with a higher level of understanding of physiological processes—thus providing a richer, denser, and more interconnected mental landscape of qualitative and quantitative checkpoints. However, our study also identified students who developed a high level of confidence in incorrect solutions to physiological problems over the course of the seminar series, which hints at preconceptions or even misconceptions. This provides a new starting point for targeted interventions as well as for a critical assessment focused on which resources and strategies these students utilized.

## Data Availability Statement

The raw data supporting the conclusions of this article will be made available by the authors, without undue reservation.

## Ethics Statement

Ethical review and approval was not required for the study on human participants in accordance with the local legislation and institutional requirements. The participants provided their written informed consent to participate in this study.

## Author Contributions

JR provided the idea for this study, developed the instrument for knowledge assessment, and was involved in the data collection and in preparing and reviewing the manuscript. JR-S conducted the analyses and co-wrote the manuscript. OZ-T developed, in collaboration with JR, the study design, the instrument for confidence testing, was involved in the analyses, and co-wrote the manuscript. VK and MW were involved in the development of the instrument for knowledge assessment and the data collection and supported the analyses. M-TN was involved in the organization of the data collection and supported the analyses. All authors contributed to the article and approved the submitted version.

## Conflict of Interest

The authors declare that the research was conducted in the absence of any commercial or financial relationships that could be construed as a potential conflict of interest.

## Publisher’s Note

All claims expressed in this article are solely those of the authors and do not necessarily represent those of their affiliated organizations, or those of the publisher, the editors and the reviewers. Any product that may be evaluated in this article, or claim that may be made by its manufacturer, is not guaranteed or endorsed by the publisher.

## Footnotes

1 At each of the 3-h seminars, up to six physiology topics were discussed in a group comprising 20 students and a lecturer. Each topic was introduced by one of the students for about 10 min. The lecturer evaluated the quality of the presentation (satisfactory/non-satisfactory). To successfully complete this seminar series, which also includes two 30-question MC examinations, students were not allowed to have more than one non-satisfactory performance.

2 Though the measured contents of physiology also involved procedural and conditional knowledge facets, the items in this test are not designed to explicitly assess them in a valid and reliable way.
