# Supplementary material for: Patterns of Domain-Specific Learning Among Medical Undergraduate Students in Relation to Confidence in Their Physiology Knowledge: Insights From a Pre–post Study
Source: Front Psychol. 2022 Feb 10;12:562211. doi: 10.3389/fpsyg.2021.562211 (PMC8867175; doi:10.3389/fpsyg.2021.562211)
Supplement: Supplementary file 1 [file Data_Sheet_1.pdf]

Supplementary Material

Table S.1. Differences between profiles: low confidence vs. medium confidence vs. high confidence at t1.

	low confidence	medium confidence	high confidence		Tukey post-estimation			
	M ± *SD* (n) / %	*M* ± *SD* (n) / %	*M* ± *SD* (n) / %	*F /chi², p*	*p* low vs medium	*p* medium vs high	*p* low vs high	ω²/Cramer`s *V*
Confidence level at t1	1.51 ± 0.193 (41)	2.11 ± 0.182 (71)	2.65 ± 0.127 (13)	245.36, < 0.001	< 0.001	< 0.001	< 0.001	0.796
Confidence level at t2	2.52 ± 0.404 (35)	2.83 ± 0.408 (47)	2.81 ± 0.526 (42)	5.60, 0.0047	0.008	0.984	0.015	0.069
Knowledge test score at t1	4.49 ± 1.660 (41)	4.141 ± 1.397 (71)	4.23 ± 2.069 (22)	0.61, 0.542	0.514	0.973	0.812	-0.006
Knowledge test score at t2	7.59 ± 2.166 (37)	7.98 ± 1.974 (49)	7.52 ± 2.074 (46)	0.67, 0.515	0.669	0.528	0.986	-0.005
Knowledge test difference score	3.00 ± 2.008 (37)	3.63 ± 2.007 (49)	4.18 ± 2.089 (11)	1.81, 0.170	0.333	0.700	0.215	0.016
Learning motivation[2]	2.14 ± 0.582 (37)	2.16 ± 0.770 (49)	1.94 ± 0.652 (46)	1.41, 0.248	0.987	0.267	0.400	0.006
Intelligence test score	13.46 ± 3.529 (41)	12.17 ± 3.304 (71)	11.55 ± 3.555 (22)	2.82, 0.063	0.134	0.735	0.089	0.026
UEQ[1] grade	1.39 ± 0.488 (37)	1.54 ± 0.563 (49)	1.41 ± 0.557 (45)	1.04, 0.357	0.415	0.465,	0.988	0.001
Age	21.22 ± 3.001 (37)	22.86 ± 4.632 (49)	21.73 ± 2.618 (44)	2.39, 0.096	0.096	0.290	0.801	0.021
Sex, male	17.07 %	30.99 %	57.14 %	3.526, 0.172	-	-	-	0.162
Language, German	92.68 %	85.92 %	77.27 %	2.979, 0.225	-	-	-	0.149
Medical vocational training, yes	9.76 %	26.76 %	4.55 %	8.309, 0.016	-	-	-	0.249
Advanced course in school in biology, yes	45.95 %	59.18 %	52.17 %	1.504, 0.471	-	-	-	0.107
Advanced course in school in chemistry, yes	18.92 %	24.49 %	28.26 %	0.977, 0.614	-	-	-	0.086
Advanced course in school in physics, yes	8.11 %	4.26 %	21.74 %	7.885, 0.019	-	-	-	0.244
Advanced course in school in maths, yes	51.35 %	46.94 %	43.48 %	0.510, 0.775	-	-	-	0.062
Advanced course in school in sciences, no	21.62 %	16.33 %	30.43 %	2.725, 0.256	-	-	-	0.144

Note. [1]UEQ = university entrance qualification. In Germany, lower numbers indicate better grades (1 to 6). [2] Inverted scale, lower numbers indicate higher motivation score. $\omega^2 < 0.01$ = very small, 0.01-0.06 = small, 0.06-0.14 = medium, and >0.14 = large. Cramer`s *V* < 0.1 = negligible, 0.1-0.29 = small, 0.3-0.49 = medium and ≥ 0.5 = large effect.

Table S.2. Differences between profiles: low confidence vs. medium confidence vs. high confidence at t2.

	low confidence	medium confidence	high confidence		Tukey post-estimation			
	M ± *SD* (n) / %	*M* ± *SD* (n) / %	*M* ± *SD* (n) / %	*F* /*chi²*, *p*	*p* low vs medium	*p* medium vs high	*p* low vs high	ω²/Cramer`s *V*
Confidence level at t1	1.88 ± 0.336 (6)	1.69 ± 0.354 (28)	2.07 ± 0.382 (91)	10.95, < 0.001	0.518	< 0.001	0.452	0.137
Confidence level at t2	1.81 ± 0.184 (10)	2.37 ± 0.149 (37)	3.03 ± 0.270 (77)	184.38, < 0.001	< 0.001	< 0.001	< 0.001	0.747
Knowledge test score at t1	3.17 ± 0.753 (6)	4.48 ± 1.573 (29)	4.26 ± 1.626 (99)	1.70, 0.186	0.159	0.790	0.233	0.010
Knowledge test score at t2	5.60 ± 2.066 (10)	7.08 ± 1.673 (37)	8.24 ± 1.992 (85)	11.28, < 0.001	0.080	0.007	< 0.001	0.135
Knowledge test difference score	2.50 ± 2.345 (6)	2.59 ± 1.842 (29)	3.95 ± 1.987 (62)	5.52, 0.005	0.995	0.007	0.201	0.085
Learning motivation[2]	2.25 ± 0.486 (10)	2.12 ± 0.567 (37)	2.04 ± 0.747 (85)	0.55, 0.576	0.859	0.799	0.618	-0.007
Intelligence test score	12.83 ± 4.535 (6)	12.93 ± 3.240 (29)	12.30 ± 3.480 (99)	0.40, 0.670	0.998	0.669	0.930	-0.009
UEQ[1] grade	1.79 ± 0.806 (10)	1.42 ± 0.535 (37)	1.42 ± 0.499 (84)	2.16, 0.119	0.139	0.105	1.000	0.017
Age	22.00 ± 4.000 (10)	22.11 ± 4.033 (37)	21.96 ± 3.459 (83)	0.02, 0.980	0.996	0.978	1.000	-0.015
Sex, male	16.67 %	13.79 %	32.32 %	4.230,0.121	-	-	-	0.178
Language, German	16.67 %	6.90 %	15.15 %	1.371, 0.504	-	-	-	0.101
Medical vocational training, yes	66.67 %	89.66 %	80.81 %	2.210, 0.331	-	-	-	0.128
Advanced course in school in biology, yes	40 %	54.05 %	54.12 %	0.738, 0.692	-	-	-	0.075
Advanced course in school in chemistry, yes	20 %	13.51 %	29.41 %	3.654, 0.161	-	-	-	0.166
Advanced course in school in physics, yes	10 %	5.41 %	14.12 %	1.963, 0.375	-	-	-	0.122
Advanced course in school in maths, yes	40 %	37.84 %	51.76 %	2.218, 0.330	-	-	-	0.130
Advanced course in school in sciences, no	30 %	27.03 %	20 %	1.051, 0.591	-	-	-	0.089

Note. [1]UEQ = university entrance qualification. In Germany, lower numbers indicate better grades (1 to 6).[2] Inverted scale, lower numbers indicate a higher motivation score ω²< 0.01 = very small, 0.01-0.06 = small, 0.06-0.14 = medium, and >0.14 = large. Cramer`s *V* < 0.1 = negligible, 0.1-0.29 = small, 0.3-0.49 = medium, and ≥ 0.5 = large effect.
